# Characterization of the role of Facebook groups for patients who use scalp cooling therapy: a survey study

**DOI:** 10.1007/s00520-024-08534-y

**Published:** 2024-05-15

**Authors:** Lucy Rose, Madison Novice, Sonja Kobayashi, Abena Minta, Taylor Novice, Kristen Lo Sicco, Brittany Dulmage

**Affiliations:** 1 Present Address: The Ohio State University College of Medicine, Columbus, OH USA; 2 Present Address: The University of Michigan Medical School, Ann Arbor, MI USA; 3 Department of Dermatology, Henry Ford Health System, Detroit, MI USA; 4 Department of Dermatology, NYU Grossman School of Medicine, The Ronald O. Perelman, New York City, NY USA; 5 Department of Dermatology, The Ohio State University Wexner Medical Center, Columbus, OH USA

**Keywords:** Alopecia, Chemotherapy-induced alopecia, Hair loss, Oncodermatology, Survivorship

## Abstract

Since the emergence of scalp cooling therapy (SCT) for the prevention of chemotherapy-induced alopecia (CIA), support groups on social media platforms for interested patients have surfaced. Though there are over 20,000 active members across SCT Facebook groups, little is known about how members use this platform. A 23-question survey was posted in five scalp cooling Facebook groups, reaching 219 women. Results indicated that these Facebook groups play clear roles in providing the following: (1) a supportive community for patients, (2) instructions for SCT use, (3) advice regarding insurance coverage and reimbursement, and (4) recommendations for over-the-counter products for hair loss. Despite reported interest in hair loss products, only 5% of patients sought medical treatment from dermatologists. Due to group-specific access restrictions, private Facebook groups provide patients with a protected platform to learn more about SCT from both those with personal experience and SCT company specialists. Providers may consider recommending these online groups to interested patients during the scalp cooling counseling process. As patients with CIA express a growing interest in over-the-counter hair, eyebrow, and eyelash products, it is important for dermatologists to be aware of where their patients obtain recommendations, and further, if these recommendations have clinical evidence of efficacy.

**Supplementary Information:**

The online version contains supplementary material available at 10.1007/s00520-024-08534-y.

## Introduction

Patients diagnosed with cancer may turn to social media platforms while navigating their new medical diagnosis. Online groups can provide emotional support from people going through similar experiences, advice on various cancer-related products to use, research updates, and answers to questions regarding potential treatments and associated side effects. Patients with breast cancer in particular are increasingly using these online platforms [1]. A qualitative study found that online support groups offer safe spaces for patients to discuss sensitive topics [2]. There is even evidence that engagement with online support systems has positive effects for patients. A randomized control trial (RCT) found that breast cancer survivors who receive regular contact on message boards and mailing lists related to breast cancer survivorship had an improved quality of life (QoL) score, as evidenced by a lesser degree of depression, stress, and trauma [3–5].

Hair loss secondary to chemotherapy is a common cause of distress in patients with cancer. Women cited alopecia as the single most psychologically upsetting adverse effect of chemotherapy [6]. Psychological stress secondary to hair loss presents as decreased self-esteem, and lower scores on body image and perceived attractiveness surveys [7]. As scalp hair is recognized as an integral part of personal identity, techniques for preventing hair loss while undergoing chemotherapy have emerged.

Scalp cooling therapy (SCT) or scalp hypothermia is a technique used to mitigate alopecia in patients undergoing chemotherapy for cancer treatment. For patients who elect to use SCT while undergoing chemotherapy, one option is a dynamic cooling machine in which they wear a cap before, during, and after chemotherapy with coolant that is being circulated through a temperature-regulated device. The other option is manual cold capping, which requires a “capper” (i.e., friend, family member or trained professional) to change subfreezing caps every 25 min to keep the scalp cool. SCT has demonstrated promising results, with ranges of 50–90% of women not wearing cranial prosthetics or head coverings at follow-up visits, as well as higher percentages of excellent scores on the Dean scale (used for quantification of chemotherapy-induced alopecia), indicating less than 25% hair loss [8, 9].

Since the invention of SCT for the prevention of hair loss, Facebook groups have emerged for patients who are using scalp cooling or are interested in learning more about it. Machine scalp cooling companies, Paxman^TM^ and DigniCap^TM^, have created Facebook groups for people interested in discussing these products. Facebook groups for patients using manual cold caps also exist, though were not created by companies. With over 20,000 total members between various scalp cooling and cold cap Facebook groups, it is evident that members are using this platform. However, it is unclear how members are specifically using these Facebook groups.

## Methods

This study was deemed IRB exempt. An online survey consisting of 23 questions **(**see supplement**)** was administered to members of five Facebook groups titled: DigniCap, Chemo Cold Cappers Support Group, Paxman Scalp Cooling Group, Chemo Cold Caps, and the Paxman Scalp Cooling Group in Columbus, Ohio. As of September 1, 2023, these groups had approximately 1500 members, 5441 members, 9047 members, 1330 members, and 247 members, respectively (Table 1). Researchers asked moderators of the Facebook groups for permission prior to posting the survey link in each group or if moderators preferred to post the surveys themselves. A total of six posts were made in each Facebook group over the course of five weeks.

**Table 1:** Facebook group descriptions

Facebook group name	Number of members (as of 9/1/2023)	Post frequency	Who created and moderates the group	Group description
DigniCap Connect	1500	58 posts/month	DigniCap (company)	“This group is for people who have used or are planning to use The DigniCap Scalp Cooling System. Here you can connect with people going through similar experiences and share information and advice.”
Chemo Cold Cappers Support Group	5441	252 posts/month	Former patients	“I wanted to create this group in order for us to share our experience with our very own cold capping and create a space for those that will be cold capping to ask questions and get advice. As we all know, there is not a lot of information out there about capping and I think it would be great for us to be able to share what we know and what we have learned along the way.” “You are welcome to add fellow cold cappers to the group, but they MUST have either used cold caps as part of treatment, or will be using caps as part of treatment. - No exceptions, and please no companies.”
Paxman Scalp Cooling Group	9047	437 posts/month	Paxman (company)	“Welcome to the Paxman Scalp Cooling Group - a place to talk all things scalp cooling, and find advice and support from people who have been through the process.”
Chemo Cold Caps	1330	37 posts/month	Former patients	“This group is intended to educate and support those using or interested in using cold caps during chemo treatment.”
Paxman Scalp Cooling Group in Columbus, Ohio	247	Has since been deactivated.	Former patients	Has since been deactivated.

Potential study participants included all members of the private Facebook groups. Inclusion criteria included (1) age greater than or equal to 18 years and (2) past or current use of SCT for the prevention of chemotherapy-induced alopecia. All racial and ethnic subgroups were included.

Survey questions referred to participant interaction within the Facebook group. No survey questions were related to participant interaction with scalp cooling companies or hair product companies. Survey questions were designed based on the research team’s experience with and interest in specific topics/outcomes regarding SCT (see supplement**).** Previously validated scales were not used when creating the survey.

Descriptive statistics were used to report baseline patient characteristics. We utilized chi-square tests on Microsoft Excel for outcome comparisons (e.g., insurance coverage (yes/no) based on scalp cooing type). *P*-values of <0.05 were considered statistically significant.

## Results

### Demographics

A total of 219 women completed the online survey. Of the 219 women, 154 (71%) used a scalp cooling machine, while 63 (29%) used manual cold caps. The mean age of patients when they started SCT was 50 years, with a range of 25 to 74 years of age. A majority of women identified as White (92.2%), 11 were Asian, five were African American or Black, and two were American Indian or Alaska Native. Eleven (5.3%) women identified as Hispanic or Latino. Most women reported having private health insurance (79%), with Medicare (9%), other (6%), no insurance (5%), and Medicaid (1%) being less common (Table 2).

**Table 2:** Patient demographics

	*N (%)*
Gender	
Female	219 (100)
Race	
Hispanic/Latino	11 (5.3)
White	200 (92.2)
Asian	11 (5.1)
African American or Black	5 (2.3)
American Indian or Alaska Native	2 (0.9)
Age	
Below age 31 Between 31 and 40	6 (2.7) 32 (14.8)
Between 41 and 50	75 (34.7)
Between 51 and 60	66 (30.5)
Greater than or equal to 61	36 (16.7)
Insurance status	
Private	173 (79.0)
Medicare	20 (9.1)
Other	13 (5.9)
None	11 (5.0)
Medicaid Type of SCT Machine scalp cooling Manual cold caps	2 (0.9) 154 (71.0) 63 (29.0)

### Cost of scalp cooling

When asked the degree to which cost served as a financial barrier to SCT use, participants responded the following: little to none (52.5%), moderate (37.9%), significant (9.6%). One hundred and four individuals** (**47.5%) attempted to obtain insurance coverage or reimbursement for SCT. Of those who attempted, only 11.7% received full coverage, while 11.7% received partial coverage. There was not a significant statistical difference regarding insurance coverage based on type of scalp cooling device (*p* = 0.23); specifically, between those who had used scalp cooling machines and had received full or partial coverage versus those who used manual cold caps and received full or partial coverage.

### Motivation for joining a Facebook group

Reasons for joining a SCT-specific Facebook group included (1) to gain support from others going through a similar experience (193/219**,** 88.1%), (2) to receive SCT instructions (180/219, 82.2%), (3) to learn about prior patients’ SCT outcomes (success/failure) (153/219, 69.9%), to obtain advice on (4) hair regrowth products (143/219, 65.3%) or (5) camouflaging techniques (e.g., wigs, topical camouflaging agents) (101/219, 46.1%), and (6) to learn about the finances of SCT (53/219, 24.2%).

### Scalp cooling instructions

When asked about their primary source for instructions for how to scalp cool while undergoing chemotherapy infusions, most used their scalp cooling company’s website (88.5%), while others used Facebook groups (49.1%), YouTube (20.2%), and their oncologists (18.2%). Most women (80%) had posted about or commented on a Facebook post related to SCT instructions in a Facebook group.

### Hair loss management

Based solely on Facebook reviews, participants were most likely to have tried over-the-counter hair loss vitamins or supplements (21.8%), cranial prosthetics or hair pieces (18.0%), hair growth shampoos and conditioners (17.1%), and Toppik (14.2%), which is a hair loss camouflaging spray with fibers that cling to remaining hair. Despite seeking out hair regrowth product information in Facebook groups, only 5% of patients had seen a dermatologist for hair loss concerns during or after chemotherapy.

### Eyebrow and eyelash loss

Participants were more likely to turn to Facebook groups (71.3%) instead of oncologists (9.9%), or dermatologists (4.0%) for eyebrow and eyelash loss product recommendations. Thirty-three percent reported posting in a Facebook group asking for product recommendations for eyebrow and eyelash loss. Of the eyebrow products used, 42.5% had used eyebrow pencils, 16.9% had used eyebrow powder, 15.9% had tried microblading, 14.4% had used Latisse (generic bimatoprost), and 3.5% had permanent eyebrow tattoos. For eyelash loss, an eyelash growth serum was the most used (22.4%), followed by Latisse (bimatoprost) (16.9%), and less commonly false lashes (13.9%). Only 6% had seen a dermatologist for the evaluation of eyebrow or eyelash loss during or after chemotherapy.

## Discussion

### Takeaway 1: Patients use Facebook groups for support from others going through similar experiences

Most individuals in our study joined a scalp cooling Facebook group to receive support from other patients going through a similar experience. Patients recently diagnosed with cancer may turn to various support networks within their communities. In recent years, social networking sites such as Facebook have provided a platform for cancer patients seeking to connect and share experiences. These patient-centered groups provide a unique avenue for individuals to share their personal journeys, discuss treatment options, and exchange insights on managing the challenges associated with cancer diagnosis and treatment [10]. Researchers have found that the main focus of these groups included disease awareness, disease prevention, and support for patients and their families [11]. In particular, breast cancer groups have been shown to increase patient education, communication, engagement and patient empowerment [12, 13]. The impact of engaging with these social media groups has been established, with one study demonstrating that engagement augments self-efficacy and psychological wellbeing and leads to decreased symptom distress [14].

Interestingly, the ability to share personal stories while also being able to support others has been shown to provide reciprocal benefits. Breast cancer patients who received higher levels of support on social media had reduced breast cancer related concerns [15]. Simultaneously, those who supported others going through similar experiences were found to have increased positive coping strategies and a greater ability to reframe their own hardships into a more positive light [15]. Thus, Facebook groups provide a unique platform for patients to share their experiences, increase their support network, and create a community, which in turn, improves their own QoL.

### Takeaway 2: Patients use Facebook groups to learn about SCT instructions

Almost half of participants used the Facebook group as a source for SCT instructions, and nearly 80% reported posting or commenting on posts regarding SCT instructions. Though scalp cooling companies, such as Paxman^TM^, Penguin^TM^, and DigniCap^TM^, have patient instructions on their websites (https://coldcaphaircare.com/category/guides/, https://penguincoldcaps.com/faqs/, https://dignicap.com/patient-instructions/.) , we found that patients also benefit from having a means to ask questions in an informal setting and among people who have similar experiences.

Facebook groups offer a platform for asking questions to both others who have personal experience using SCT and company-employed specialists. In Facebook groups created by SCT companies themselves, company employees have the means to directly comment on patient questions. Company employees can provide expertise on subjects by linking guides, tutorials, and blog posts. Non-company members can also provide advice from their personal experiences. Group members are able to attach pictures and links to products that they feel have been beneficial for them.

### Takeaway 3: Patients use Facebook groups to obtain information and advice for achieving insurance coverage for SCT costs

Individuals reported joining their respective Facebook groups to learn more about SCT cost. Of the participants who attempted to obtain insurance coverage/reimbursement, only 11.7% received full coverage and 11.7% received partial coverage. Almost half of participants reported cost being a barrier to SCT use; however, it is important to note that, given the well-known association between race and socioeconomic status, our predominately White patient population likely *underestimates* SCT’s overall financial burden in certain populations [16].

Our findings are consistent with previous literature demonstrating the financial burden posed by scalp cooling [17–19]. Without financial assistance, Paxman^TM^ and DigniCap^TM^ estimate that SCT costs, on average, $1500–$3000 per chemotherapy course. For patients who use manual cold caps, costs of dry ice and coolers are added. While two CPT codes were released following FDA clearance of DigniCap^TM^ and Paxman^TM^ machine systems in 2015 and 2017, respectively, insurance coverage is still not routine. In addition, no codes unique to manual caps have been assigned [12]. A national survey on oncologist practice patterns with scalp cooling found that cost was the primary reason providers did not recommend scalp cooling to their patients [20]. Moreover, patients who use scalp cooling are more likely to have private insurance or live in zip codes with average income greater than $100,000 [21–23]. Local and national non-profit organizations have emerged to combat this inequity in care by providing need-based financial assistance, but there is still a push for more comprehensive insurance coverage of scalp cooling.

As many participants turned to Facebook groups for advice on cost management of scalp cooling, our data highlights how these platforms can serve as an important educational resource for providers to recommend to patients as they counsel them on the option of SCT.

### Takeaway 4: Patients using SCT turn to Facebook groups for hair, eyebrow, and eyelash product recommendations

In our study, 65% and 46% of patients used Facebook groups to seek advice for hair regrowth and hair loss camouflage products, respectively. Because these private Facebook groups are meant for patients and not board-certified clinicians, posts regarding product recommendations may not fully consider the potential side effects as well as their clinical efficacy in patients with cancer. Here, we analyze the products commonly mentioned in Facebook groups to better understand if they are safe and beneficial.

#### Vitamins and supplements

Of the Facebook group-recommended products patients used, hair loss vitamins and supplements were the most popular. In recent years, nutritional supplements and vitamins have become popular tools for promoting hair regrowth. Nutraceuticals (i.e., natural dietary and botanical supplements) have minimal evidence to support their use for treatment of hair loss, and physicians may be hesitant to recommend them due to fear of a potential interaction with cancer treatments [24]. In Facebook groups, commonly mentioned nutraceuticals include Nutrafol, Viviscal, and biotin. These products are not FDA approved and are not required to undergo as rigorous of testing prior to distribution. Therefore, in Table 3, we review of these treatments so that both oncologists and dermatologists can counsel patients on their safety profiles and results from clinical trials.

**Table 3:** Review of hair growth vitamin and supplements recommended in scalp cooling Facebook groups

Product	Study	Design	Duration	Results	Clinical considerations
Nutrafol	Ablon and kogan, 2018 [25]	Randomized, double-blind, placebo-controlled study	Daily for 6 months	Increase in terminal and vellus hairs by day 90 and 180 vs. placebo (*p* = <0.009); improvement in hair growth (*p* = 0.016) and overall hair quality (*p* = 0.005)	No data on use in patients with cancer undergoing treatment. Contains Palmetto, which is a plant-based DHT* blocker.
	Stephens et al., 2022 [26]	Prospective, single-blind study	Daily for 6 months	83.7% of men and 79.5% of women had significant increases in hair appearance, volume, and scalp coverage, as rated by investigators	
ViviScal®	Bloch et al., 2017 [27]	Controlled, monocentric study	Daily for 6 months in patients with telogen effluvium	Increase in hair follicle number in 83%	Limited evidence only in patients with telogen effluvium.
	Glynis, 2012 [28]		Daily for 6 months	No significant change in vellus hairs between treatment and placebo	
Biotin	Patel et al., 2017 [29]	Systematic review on biotin efficacy in hair and nail growth that included 18 case reports of 18 patients. No clinical trials were found.	Doses and duration varied by case report	Some degree of improvement in hair growth or nail growth after 10 days to 6 months of treatment	- Evidence only in populations with pathology for either poor hair or nail growth (e.g., uncombable hair syndrome, valproic acid-induced alopecia) - Can interfere with laboratory immunoassays (e.g., concentration of immunosuppressive drugs, thyroid function tests, cardiac enzymes, hepatitis, vitamin d) [30–33] - AAD** recommended discontinuation of biotin for promotion of hair regrowth (https://dignicap.com/patient-instructions/) [34]

^*^DHT dihydrotestosterone

^**^AAD American Academy of Dermatology

#### Camouflage techniques

When asked why they joined SCT Facebook groups, 47% of women reported wanting to learn more about hair camouflaging options. Camouflaging techniques have been well-received in patients looking for non-permanent, non-systemic ways of hiding areas of hair loss. For hair on the scalp, camouflaging options include cranial prosthetics and hair pieces, pigmented concealing powders, lotions, and sprays. Advantages of these non-permanent products include ease of removal, extensive hair color and style options, and lack of interference with systemic medications. Disadvantages include required daily application and that these topical fibers only attach to remaining hair on the scalp, but not in areas that are devoid of hair. In Table 4, we review the camouflage techniques mentioned by participants in our study, including important clinical considerations for providers when counseling patients on these options.

**Table 4:** Review of camouflage techniques for hair, eyebrow, and eyelash loss recommended in Facebook groups

Treatment	Survey results* (*n*, %)	Mechanism	Permeant (yes or no)	Clinical considerations
Toppik	*n*, 17%	Topical spray containing particles of wool keratin that adhere to remaining hair fibers on the scalp, making hair appear thicker [35].	No	May not adhere as well to areas where no hair is growing, such as in chemotherapy-induced alopecia. 92.5% of participants noted a high level of satisfaction, as evidenced by increased levels of confidence and perceived attractiveness [36].
False eyelashes	*n*, 12.4%	n/a	No	- Can be difficult to apply in absence of normal eyelashes - Can lose whatever eyelashes are left when taking off the false lashes - Glue may irritate skin
Eyebrow pencils	40%	Over-the-counter cosmetic products	No	N/A
Eyebrow powder	16.8	Over-the-counter cosmetic products	No	N/A
Microblading or eyebrow tattoos	16.1 + 2.2	Pigment is deposited in the papillary dermis using a manual device and blade consisting of stacked needles [37, 38].	Semi-permanent	Increased risk of infection in cancer patients (https://www.pmuhub.com/microblading-for-cancer-patients-realistic-brow-reconstruction/) .

^*^Number of patients who decided to use the given product based on Facebook reviews

### Takeaway 5: Despite patients reporting hair, eyebrow, and eyelash loss concerns during and after scalp cooling, they are not utilizing dermatologists or oncologists for treatment advice

Supportive oncodermatology is a newer field that aims to decrease the morbidity associated with dermatologic adverse effects (dAE) from cancer treatments. These dermatologic sequelae can lead to interruptions and modifications of life-saving cancer treatments [39, 40]. Studies have shown that patients who experience long-term hair loss after chemotherapy treatments score low on QoL measures [41]. In addition to hair loss on the scalp, loss of eyebrows and eyelashes can also contribute to emotional distress [42]. Fortunately, a survey study found that after receiving supportive treatment for dAEs from a dermatologist, patients experienced a modest improvement in QoL, as evidenced by increased Dermatology Life Quality Index (DLQI) and Patient Satisfaction Questionnaire (PSQ-18) scores [43].

Only 6% of survey participants had seen a dermatologist for hair loss or eyebrow/eyelash loss. This finding reflects a major incongruity between those interested in receiving alopecia treatment and those who present to dermatologists for clinical evaluation and medical treatment. Though there is evidence that patients with cancer therapy-induced alopecia benefit from dermatologic care, there is a need for future studies to characterize barriers in how often patients are referred to dermatologists and how accessible dermatologists are for patients with cancer.

## Conclusion

This survey study demonstrated that private Facebook groups provide a supportive community for patients, instructions for SCT, advice for insurance coverage and reimbursement, and hair, eyebrow, and eyelash loss product recommendations from others who are also navigating SCT. Providers may therefore consider recommending this platform to patients who want to learn more about SCT and/or receive peer support through the process. At the same time, providers should also emphasize that any medical and/or product advice given in these groups may not be consistent with their recommendations. As patients who experience CIA express a strong interest in hair loss product recommendations, it is important for future studies to focus on how patients can obtain safe and effective treatment options from clinical experts.

### Supplementary Information

Below is the link to the electronic supplementary material.

Supplementary file1 (DOCX 18 KB)

## References

[1] Davison KP, Pennebaker JW, Dickerson SS (2000). Who talks? The social psychology of illness support groups. Am Psychol..

[2] Sharf BF (1997). Communicating breast cancer on-line: support and empowerment on the Internet. Women Health..

[3] Gustafson DH, Hawkins R, Pingree S, McTavish F, Arora NK, Mendenhall J, Cella DF, Serlin RC, Apantaku FM, Stewart J, Salner A (2001). Effect of computer support on younger women with breast cancer. J Gen Intern Med.

[4] Gustafson DH, McTavish FM, Stengle W, Ballard D, Hawkins R, Shaw BR, Jones E, Julèsberg K, McDowell H, Chen WC, Volrathongchai K, Landucci G (2005). Use and impact of eHealth system by low-income women with breast cancer. J Health Commun..

[5] Winzelberg AJ, Classen C, Alpers GW, Roberts H, Koopman C, Adams RE, Ernst H, Dev P, Taylor CB (2003). Evaluation of an internet support group for women with primary breast cancer. Cancer..

[6] McGarvey E, Baum L, Pinkerton Relana, Rogers L (2008). Psychological sequelae and alopecia among women with cancer. Cancer Practice.

[7] Mundstedt K, Manthey N, Sachsse S, Vahrson H (1997). Changes in self-concept and body image during alopecia induced chemotherapy. Support Care Cancer.

[8] Van den Hurk J, Peerbooms M, van de Poll-Franse L, Nortier J, Coebergh J, Breed W (2012). Scalp cooling for hair preservation and associated characteristics in 1411 chemotherapy patients - results of the Dutch Scalp Cooling Registry. Acta Oncol.

[9] Cigler T, Isseroff D, Fiederlein B, Schneider S, Chuang E, Vahdat L, Moore A (2015). Efficacy of scalp cooling in preventing chemotherapy-induced alopecia in breast cancer patients receiving adjuvant docetaxel and cyclophosphamide chemotherapy. Clin Breast Cancer.

[10] Bender JL, Jimenez-Marroquin MC, Jadad AR (2011). Seeking support on facebook: a content analysis of breast cancer groups. J Med Internet Res..

[11] De la Torre-Díez I, Díaz-Pernas FJ, Antón-Rodríguez M (2012). A content analysis of chronic diseases social groups on Facebook and Twitter. Telemed J E Health.

[12] Falisi AL, Wiseman KP, Gaysynsky A, Scheideler JK, Ramin DA, Chou WS (2017). Social media for breast cancer survivors: a literature review. J Cancer Surviv..

[13] Aristokleous I, Karakatsanis A, Masannat YA, Kastora SL (2023). The role of social media in breast cancer care and survivorship: a narrative review. Breast Care (Basel)..

[14] Zhu J, Ebert L, Wai-Chi Chan S (2017). Integrative review on the effectiveness of internet-based interactive programs for women with breast cancer undergoing treatment. Oncol Nurs Forum..

[15] Kim E, Han JY, Moon TJ, Shaw B, Shah DV, McTavish FM, Gustafson DH (2012). The process and effect of supportive message expression and reception in online breast cancer support groups. Psychooncology..

[16] Williams DR, Priest N, Anderson NB (2016). Understanding associations among race, socioeconomic status, and health: patterns and prospects. Health Psychol..

[17] Shaw JM, O’Brien J, Chua S (2018). Barriers and enablers to implementing scalp cooling in Australia: a qualitative study of health professionals’ attitudes to and experience with scalp cooling. Support Care Cancer.

[18] Peerbooms M, van den Hurk CJ, Breed WP (2015). Familiarity, opinions, experiences and knowledge about scalp cooling: a Dutch survey among breast cancer patients and oncological professionals. Asia Pac J Oncol Nurs.

[19] Shaw J, Baylock B, O’Reilly A (2016). Scalp cooling: a qualitative study to assess the perceptions and experiences of Australian patients with breast cancer. Support Care Cancer.

[20] Rapunzel Project (2021) Accessed April 12, 2023. http://www.rapunzelproject.org/ColdCaps/TheCaps.aspx

[21] Novice T (2022). Factors influencing scalp cooling discussions and use at a large academic institution: a single-center retrospective review. Supportive Care in Cancer.

[22] Singer S, Tkachenko E, Sharma P, Nelson C, Mostaghimi A, LeBoeuf NR (2020). Geographic disparities in access to scalp cooling for the prevention of chemotherapy-induced alopecia in the United States. J Am Acad Dermatol.

[23] Rose L, Schnell PM, Radcliff L, Lustberg M, Dulmage B (2023). Retrospective cohort study of scalp cooling in breast cancer patients. Supportive Care in Cancer.

[24] Dell'Acqua G, Richards A, Thornton MJ (2020). The potential role of nutraceuticals as an adjuvant in breast cancer patients to prevent hair loss induced by endocrine therapy. Nutrients..

[25] Ablon G, Kogan S (2018). A six-month, randomized, double-blind, placebo-controlled study evaluating the safety and efficacy of a nutraceutical supplement for promoting hair growth in women with self-perceived thinning hair. J Drugs Dermatol..

[26] Stephens TJ, Berkowitz S, Marshall T, Kogan S, Raymond I (2022). A prospective six-month single-blind study evaluating changes in hair growth and quality using a nutraceutical supplement in men and women of diverse ethnicities. J Clin Aesthet Dermatol.

[27] Bloch LD, Escudeiro CC, Sarruf FD (2017). Efficacy assessment of a nutraceutical with a marine protein complex in the reduction of female telogen effluvium. J Dermat Cosmetol..

[28] Glynis A (2012). A double-blind, placebo-controlled study evaluating the efficacy of an oral supplement in women with self-perceived thinning hair. J Clin Aesthet Dermatol.

[29] Patel DP, Swink SM, Castelo-Soccio L (2017). A review of the use of biotin for hair loss. Skin Appendage Disord..

[30] Kabiri P, Weiskirchen R, van Helden J (2021). The biotin interference within interference suppressed immunoassays. J Clin Lab Anal..

[31] Ylli D, Soldin SJ, Stolze B, Wei B, Nigussie G, Nguyen H, Mendu DR, Mete M, Wu D, Gomes-Lima CJ, Klubo-Gwiezdzinska J, Burman KD, Wartofsky L (2021). Biotin interference in assays for thyroid hormones, thyrotropin and thyroglobulin. Thyroid..

[32] Frame IJ, Joshi PH, Mwangi C, Gunsolus I, De Lemos JA, Das SR, Sarode R, Balani J, Apple FS, Muthukumar A (2019). Susceptibility of cardiac troponin assays to biotin interference. Am J Clin Pathol..

[33] Waqas B, Wu A, Yim E, Lipner SR (2020). A survey-based study of physician practices regarding biotin supplementation. J Dermatolog Treat.

[34] Lipner SR (2020) Update on biotin therapy in dermatology: time for a change. J Drugs Dermatol. 19(12). 10.36849/JDD.2020.4946 10.36849/JDD.2020.4946 33346513

[35] Donovan JC, Shapiro RL, Shapiro P, Zupan M, Pierre-Louis M, Hordinsky M (2012). A review of scalp camouflaging agents and prostheses for individuals with hair loss. Dermatol Online J..

[36] Babadjouni A, Juhasz M, Pham C, Csuka E, Hedayati B, Evron E, Mesinkovska NA (2022). Patient satisfaction and adverse effects following the use of topical hair fiber fillers. Int J Trichology.

[37] Marwah MK, Kerure AS, Marwah GS (2021). Microblading and the science behind it. Indian Dermatol Online J..

[38] De Cuyper C (2015). Complications of cosmetic tattoos. Curr Probl Dermatol..

[39] Nikolaou V, Voudouri D, Tsironis G, Charpidou A, Stamoulis G, Triantafyllopoulou I, Panoutsopoulou I, Xidakis E, Bamias A, Samantas E, Aravantinos G, Gogas H, Rigopoulos D, Syrigos K, Stratigos A (2019) Cutaneous toxicities of antineoplastic agents: data from a large cohort of Greek patients. Support Care Cancer 27(12):4535–4542. 10.1007/s00520-019-04751-y 10.1007/s00520-019-04751-y 30919155

[40] Barrios DM, Phillips GS, Freites-Martinez A, Hsu M, Ciccolini K, Skripnik Lucas A, Marchetti MA, Rossi AM, Lee EH, Deng L, Markova A, Myskowski PL, Lacouture ME (2020). Outpatient dermatology consultations for oncology patients with acute dermatologic adverse events impact anticancer therapy interruption: a retrospective study. J Eur Acad Dermatol Venereol.

[41] Freites-Martinez A, Chan D, Sibaud V (2019). Assessment of quality of life and treatment outcomes of patients with persistent postchemotherapy alopecia. JAMA Dermatol..

[42] Dua P, Heiland MF, Kracen AC, Deshields TL (2017). Cancer-related hair loss: a selective review of the alopecia research literature. Psychooncology..

[43] Aizman L, Nelson K, Sparks AD, Friedman AJ (2020). The influence of supportive oncodermatology interventions on patient quality of life: a cross-sectional survey. J Drugs Dermatol..

