# Supplementary material for: Characterization of the role of Facebook groups for patients who use scalp cooling therapy: a survey study
Source: Support Care Cancer. 2024 May 15;32(6):351. doi: 10.1007/s00520-024-08534-y (PMC11096238; doi:10.1007/s00520-024-08534-y)
Supplement: Supplementary file 1 — Supplementary file1 (DOCX 18 KB) [file 520_2024_8534_MOESM1_ESM.docx]

**Scalp Cooling/Cold Caps Survey Questions**

**Demographics:**

1. Do you identify as male, female, or other?
   1. Female
   2. Male
   3. Other
   4. Prefer not to answer
2. What age were you when you started scalp cooling/cold caps? (fill in the blank)
3. What is your race?
   1. African American/Black
   2. Asian
   3. Hispanic/Latino
   4. White/Caucasian
   5. Other
4. Did you use scalp cooling or cold caps?
   1. Scalp cooling
   2. Cold caps

**Insurance/Financial barrier:**

1. Was the cost of scalp cooling/cold caps a significant barrier for you?
   1. Significant
   2. Modest
   3. Very little
   4. Not at all
2. What type of insurance do you have?
   1. Medicare/Medicaid
   2. Private insurance
   3. No insurance
   4. Other
3. Did you attempt to obtain insurance coverage or reimbursement for scalp cooling/cold caps?
   1. Yes
   2. No
4. If yes, were you successful in obtaining insurance coverage or reimbursement for scalp cooling?
   1. Yes- they covered 100% of my scalp cooling costs.
   2. Yes- they covered some of my scalp cooling costs, but not all.
   3. No- they covered none of my scalp cooling costs.
5. Did you learn about potential sources for funding or advice for how to get coverage or reimbursement on this Facebook group?
   1. Yes
   2. No

**Hair Loss:**

1. What was your primary source for obtaining scalp cooling or cold cap instructions?
   1. The scalp cooling or cold cap company’s website ex) Paxman, Penguin, DigniCap, etc.
   2. Youtube
   3. The internet (Not a company website or Youtube)
   4. A Facebook group
   5. A friend or loved one
   6. A hairstylist
   7. An oncologist
   8. A dermatologist
   9. Other
2. Have you ever posted about or commented on a post about scalp cooling or cold cap instructions in this Facebook group?
   1. Yes
   2. No
3. Did you use any of the following products because of what people in a Facebook group recommended?
   1. Hair growth serums ex) Kerastase
   2. Hair growth supplements, vitamins ex) biotin, collagen, vitamin C, Nutrafol
   3. Hair growth Shampoos, conditioners ex) Vegamoure, Nioxin
   4. Liquid or foam minoxidil (Rogaine)
   5. Other
   6. I have not used any of the following treatments because of Facebook group recommendations
4. Did you see a dermatologist for hair loss during or after cancer treatments?
   1. Yes
   2. No

**Eyebrow/eyelash loss:**

1. Since your breast cancer diagnosis, how much have you experienced **loss of your eyebrows**?
   1. Not at all
   2. Mild loss (I notice, but others would not)
   3. Significant loss (I notice and so do others)
2. Since your breast cancer diagnosis, how much have you experienced **loss of your eyelashes**?
   1. Not at all
   2. Mild loss (I notice, but others would not)
   3. Significant loss (I notice and so do others)
3. Where have you read/heard about potential eyebrow/eyelash loss treatments? (Select all that apply)
   1. Internet
   2. Facebook groups
   3. Friend or loved one
   4. Hairstylist
   5. Esthetician
   6. Oncologist
   7. Dermatologist
   8. Other
   9. I have not read about potential eyebrow/eyelash loss treatments
4. Have you ever tried these treatments for **eyelash** loss? (select all that apply)
   1. Latisse
   2. False lashes
   3. Eyelash growth serums ex) Vegamour, RevitaLash, etc
   4. Other
   5. I have not used anything for eyelash loss
5. Have you ever tried these treatments for **eyebrow** loss? (select all that apply)
   1. Latisse
   2. Eyebrow pencils
   3. Eyebrow powder
   4. Microblading
   5. Permanent tattooing
   6. Other
   7. I have not used anything for eyebrow loss
6. Have you ever tried an eyebrow or eyelash loss treatment based on a recommendation from a Facebook group post?
   1. Yes
   2. No
7. Have you ever posted about or commented on a post about eyebrow or eyelash loss in this Facebook group?
   1. Yes
   2. No
8. Did you see a dermatologist for eyebrow or eyelash loss during or after cancer treatments?
   1. Yes
   2. No

**Facebook-specific**

1. What is your main purpose of being a part of a scalp cooling/cold caps Facebook group? (check all that apply)
   1. Support from people who have been through similar experiences
   2. Information about scalp cooling/cold cap instructions
   3. Information about if scalp cooling/cold caps work
   4. Information about financial support programs or advice on insurance coverage
   5. Information about products (shampoos, supplements) to use for hair regrowth
   6. Information about products that can hide hair loss ex) wigs, hair pieces, Toppik, etc.
   7. Other
